# Multiple methods findings from a systematic scoping review and qualitative interviews to inform LGBTQ+ affirming practices for nursing education

**DOI:** 10.3389/fpubh.2026.1651099

**Published:** 2026-03-09

**Authors:** Sarah MacCarthy, Korijna Valenti, Amaya Beck, Lauryn Dancy, Frank Puga, Felesia Bowen

**Affiliations:** 1 Department of Health Behavior, Center for the Study of Sexual and Gender Health, University of Alabama at Birmingham School of Public Health, Birmingham, AL, United States; 2 Division of Gerontology, Geriatrics and Palliative Care, Heersink School of Medicine, School of Public Health, University of Alabama at Birmingham, Birmingham, AL, United States; 3 Department of Acute, Chronic and Continuing Care, University of Alabama at Birmingham School of Nursing, Birmingham, AL, United States; 4 Office of Access and Engagement, University of Alabama at Birmingham School of Nursing, Birmingham, AL, United States

**Keywords:** faculty nursing education, LGBTQ+, multiple methods, qualitative interviews, systematic scoping review

## Abstract

**Background:**

Lesbian, gay, bisexual, transgender and other gender and sexual minority people (LGBTQ+) individuals face dramatic inequities in health outcomes across the US and especially those residing in the Deep South due to pervasive LGBTQ+-related stigma and discrimination. A significant barrier repeatedly noted is the lack of clinicians trained to provide LGBTQ+-affirming care. Given that nurses play a critical role in patient care, research was needed to inform the development and piloting of LGBTQ+-specific curriculum for nursing faculty.

**Methods:**

We conducted a multiple-methods study consisting of a PRISMA-ScR–guided scoping review and qualitative descriptive interviews. A systematic search of PubMed identified studies relevant to LGBTQ+ nursing education, and 62 articles met criteria for inclusion and thematic synthesis. To further contextualize these findings, we conducted individual qualitative interviews with nursing students and faculty (*n* = 21) to explore experiences with LGBTQ+-related curriculum and instructional practices.

**Results:**

Our multiple-methods results fall into two broad domains: (1) Course content (i.e., LGBTQ+-focused theories, evolving terms and concepts, empirical evidence of health inequities, and clinical considerations); (2) Considerations on how best to deliver the course (i.e., balancing hybrid modalities, varying lengths, balancing personal and lived experience among instructors, and minimal costs, and use of validated scales in combination with qualitative questions for the evaluation).

**Discussion and conclusion:**

Given the substantial number of LGBTQ+ people across the US (2024 Gallup Poll shows 9.3% of the country's population identifies as LGBTQ+), and especially in the South (home to the largest LGBTQ+ population across Census Bureau regions), we are unlikely to ‘make America healthy again' without addressing the needs of LGBTQ+ communities. To do so, structural-level change that engages the providers in our health systems, and specifically the nurses who have the most touchpoints with our patients, is needed to reconcile the promise of good health and its achievement. Our results provide a clear roadmap to educating our nursing faculty and empowering them to deliver on our collective commitment to achieve the highest attainable standard of health for all.

## 1 Introduction

Sexual and gender minority (LGBTQ+) individuals face dramatic inequities in health outcomes. Growing research documents disproportionately worse mental (e.g., depression and anxiety) (1, 2) and physical (e.g., breast, anal, and lung cancers) (3) health compared to cisgender heterosexual adults. For example, LGBTQ+ youth are 2–3 × more likely to be homeless and to attempt suicide (2), and many older LGBTQ+ individuals are isolated and struggle to identify advocates in end-of-life care (4). Furthermore, inequities are exaggerated among racially/ethnically diverse LGBTQ+ (5), especially those residing in the Deep South (due to pervasive LGBTQ+-related stigma and discrimination). Despite dramatic shifts in the political and social acceptability of LGBTQ+ people across the United States (6), progress remains tenuous in the Deep South, especially in Alabama, recently identified by the Human Rights Campaign as a ‘high priority to achieve basic equality (7).

Research has documented how LGBTQ+ populations experience significant health disparities due to a lack of culturally responsive healthcare, both nationally (8) and in the Deep South (9). Within nursing education, efforts to integrate LGBTQ+ affirming curricula remain inconsistent and often insufficient. Nurses play a pivotal role in addressing health inequities, advocating for inclusive programs and policies, and implementing patient-centered care models. But many nursing programs dedicate limited time—often fewer than 5 h—to LGBTQ+ health topics, leaving future nurses inadequately prepared to deliver equitable and inclusive care. The absence of structured, evidence-based training in LGBTQ+-affirming care perpetuates biases, restricts patient-provider communication, and contributes to adverse health outcomes for LGBTQ+ individuals.

To bridge this gap in nursing education, it is crucial to examine existing curricular frameworks, identify best practices, and explore ways to integrate LGBTQ+ content into nursing training programs. The objective of this study is to synthesize findings from a systematic scoping review and qualitative interviews with nursing educators and students to offer recommendations for improving LGBTQ+ health in nursing education.

## 2 Pedagogical frameworks

We conducted a systematic scoping review to identify core domains and didactic strategies to include in the curriculum; we supplemented the review with qualitative interviews to provide additional context. We describe our multiple methods approach below. The methods project protocol, IRB-300012345, was submitted to the Institutional Review Board and deemed exempt. Further, our multiple methods approach detailed several different theories and frameworks critical to underpinning future curriculum, these are noted in the results and summarized in Table 1 which summarizes the theoretical and conceptual frameworks informing this study. These frameworks highlight how power, identity, and structural contexts shape curricular content, teaching strategies, and learner experiences. We drew on these frameworks when interpreting themes from both the scoping review and the qualitative interviews. In the below manuscript, we have included narrative descriptions and citations for each framework.

**Table 1:** Pedagogical frameworks.

**Type of pedagogical frameworks or theory**	**Description**
**Content: SGM specific**	
Minority stress model	The Minority Stress Model outlines how individuals from marginalized groups experience stress due to societal stigma, discrimination, and prejudice. It identifies three primary stressors: external stressors (discrimination), internal stressors (internalized stigma), and expectations of rejections (anticipatory discrimination). These stressors contribute to health disparities in vulnerable populations signifying the need for coping strategies, social support, and structural changes to reduce the impact of minority stress and promote health equity.
Cultural humility framework	The Cultural Humility Framework is an approach that emphasizes continues self- reflection, lifelong learning, and awareness of power imbalances in interactions with diverse populations through an ongoing process of self-awareness and addressing personal biases. It stresses the importance of valuing the knowledge and experiences of marginalized communities. In clinical education, this framework encourages practitioners to remain humble, seek feedback, and cultivate equitable relationships with patients to promote more inclusive and effective care.
Intersectionality	Intersectionality is a framework that examines how multiple social identities - such as race, gender, sexuality, and ability - intersect to influence individual's experiences of privilege and oppression. In clinical education, this perspective is essential for understanding how overlapping factors impact patients' health experiences and outcomes, emphasizing the need for personalized, culturally competent care that address these intersecting identities.
Norm criticism framework	The Norm Criticism Framework offers a lens to examine how dominant norms—often perceived as neutral—shape exclusion and inequity in healthcare training and practice. Rather than focusing on marginalized groups, it critiques the underlying assumptions that privilege certain idented (e.g., cisgender, able-bodied, white) as the standard. By focusing on how these norms are embedded in curricula, assessments, and clinical interactions, the framework promotes critical reflection and structural change to foster more inclusive, equity-oriented clinical environments.
**Content: SGM supportive**	
Social cognitive theory	Social Cognitive Theory (SCT) focuses on the interaction of personal, behavioral, and environmental factors in the learning process. It encourages modeling, where instructors demonstrate clinical skills for students to observe and replicate, while also promoting self-efficacy through practice and constructive feedback. By highlighting outcome expectations, students gain an understanding of how their actions impact patient health, which motivates them to apply their skills effectively. Ultimately, SCT helps cultivate both clinical competence and the cognitive skills necessary for providing effective, patient-centered care.
Theory of planned behavior	The Theory of Planned Behavior (TPB) suggests that students' intentions to engage in clinical tasks are influenced by three key factors: their attitude toward the task, the perceived social pressures from peers and instructors, and their confidence in their ability to perform the task. These factors collectively influence whether a student will successfully carry out a clinical behavior. Understanding these influences can help educators shape learning environments that foster positive attitudes, reinforce supportive social norms, and build student's confidence in their clinical skills.
Social determinants of health	The Social Determinants of Health (SDH) are the conditions in which individuals live, work, and age, influencing their health outcomes and access to care. These include economic stability, education, healthcare access, environment, and social support. Understanding these factors allows clinicians to provide more effective care and help patients overcome barriers to health by building trust through empathy.
**Teaching and learning**	
Adult learning theory	Adult Learning Theory, or andragogy, outlines how adults learn differently from children based on five key assumptions: (1) self-directed learning; (2) reliance on prior experience; (3) readiness to learn linked to social roles; (4) problem-centered learning; and (5) internal motivation. These assumptions guide the development of effective educational strategies tailored to adult learners' unique needs and contexts.
Social emotional learning theory	Social Emotional Learning (SEL) Theory offers critical framework for enhancing clinician interactions with vulnerable populations. By cultivating self-awareness, self-regulation, social awareness, relationship skills, and responsible decision-making, SEL supports emotionally attuned, ethical, and culturally responsive care. Integrating competencies into clinical practice fosters trust, improves communication, and promotes equitable, patient-centered outcomes.
Narrative pedagogy framework	The Narrative Pedagogy Framework is an educational approach that utilizes storytelling and reflective dialogue to facilitate learning in clinical settings. It positions students and educators as collaborative participants in interpreting real-life narratives, encouraging thoughtful reflection on ethical dilemmas, emotional labor, and cultural differences. By emphasizing empathy, critical thinking, and relational understanding, this framework aims to equip students with the skills necessary to address the complexities of patient care.
National league for nursing/Jeffries simulation theory	The National League for Nursing (NLN)/Jeffries Simulation Theory provides a framework for integration of simulation-based learning into nursing education. It focuses on the theory that simulation, as a teaching strategy, can enhance nursing education by providing students with an opportunity to practice and develop clinical skills in a safe and controlled environment, without the risk of harm to patients.

### 2.1 Systematic scoping review

We conducted a scoping review to identify available literature related to our broad research question: what does the peer-reviewed literature say regarding LGBTQ+ health and nursing education? A protocol for conducting the review was based on an initial review of 5 articles, then further refined after piloting the protocol on an additional 5 articles. To ensure rigor and replicability of our results, we adhered to the Preferred Items for Systematic Reviews and Meta-Analyses extension for scoping reviews (PRIMSA-ScR) guidelines (10).

In collaboration with a professional reference librarian, we developed a strategy (Table 2) and searched PubMed articles from 2018 to 2025 (*n* = 558), deduplicating (*n* = 120), dually screening abstracts (*n* = 355), reviewing full text (*n* = 118) and conducting a content analysis to identify key themes emerging from the final studies included (*n* = 62). We resolved disagreements regarding articles to include and the theme emerging from them by consensus.

**Table 2:** PubMed search strategy.

**Concept**	**Keywords**	**PubMed MeSH Terms**
Sexual and gender minorities	transgender^*^[tiab] OR “two-spirit^*^”[tiab] OR transsexual^*^[tiab] OR “non-binary”[tiab] OR “gender identity”[tiab] OR “male-to-female”[tiab] OR “female-to-male”[tiab] OR “sex reassignment”[tiab] OR “gender dysphoria”[tiab] OR “trans men”[tiab] OR “trans man” OR “cross gender”[tiab] OR “gender reassignment”[tiab] OR “trans people”[tiab] OR “gender change”[tiab] OR “gender transition”[tiab] OR “trans female”[tiab] OR “trans women”[tiab] OR “trans male”[tiab] OR transman[tiab] OR transmen[tiab] OR nonbinary[tiab] OR genderqueer[tiab] OR agender[tiab] OR bigender[tiab] OR genderfluid^*^[tiab] OR pangender[tiab] OR non-gender[tiab] OR polygender[tiab] OR androgyne[tiab] OR “third gender”[tiab] OR “gender varian^*^”[tiab] OR “sexual and gender minorit^*^”[tiab] OR SGM[tiab] OR “gender minorit^*^”[tiab] OR homosex^*^[tiab] OR gay[tiab] OR gays[tiab] OR lesbian^*^[tiab] OR msm[tiab] OR wsw[tiab] OR queer[tiab] OR “sexual orientation”[tiab] OR “same gender loving”[tiab] OR “same sex attracted”[tiab] OR “same sex couple^*^”[tiab] OR “men who have sex with men”[tiab] OR “women who have sex with women”[tiab] OR bisexual^*^[tiab] OR intersex^*^[tiab] OR intersex^*^[tiab]	“Transgender Persons”[Mesh] OR “Transsexualism”[Mesh] OR “gender identity”[Mesh] OR “Sexual and Gender Minorities”[Mesh] OR “Homosexuality”[Mesh] OR “Bisexuality”[Mesh]
Nursing education	“nursing education”[tiab] OR “nursing student^*^”[tiab] OR (nurs^*^[tw] AND curricul^*^[tw])	“Education, Nursing”[Mesh] OR “Students, Nursing”[Mesh] OR “Nursing/education”[Mesh]
Combined 1 AND 2 concepts	Filters: 2018-2023 462 results November 15, 2023	Search updated December 3 2025 from Nov 16, 2023-December 31, 2025 97 results

The review included studies that met the following criteria: 12) discusses undergraduate nursing LGBTQ+ patient care criteria; (2) discusses undergraduate nursing students, faculty, or staff attitudes toward LGBTQ+ populations; (3) discusses LGBTQ+ health inequities/disparities in nursing care; (4) data was U.S.-based. Data charting and collation were jointly developed by all authors; two reviewers independently charted all data in an Excel matrix, discussing the results, and continuously updating the data chart in an iterative process. The following types of data were extracted from each article: (1) authors; (2) title; (3) study aims; (4) methods and measures; (5) population; (6) findings; (7) key recommendations. An overview of extracted studies (Table 3) summarizes key details.

**Table 3:** Overview of articles.

**No**.	**Authors**	**Title**	**Study aim**	**Methods and measures**	**Population**	**Findings**	**Key recommendations**
1	Basile et al.	Nursing Students' Perceptions, Knowledge, and Experience in Providing Care to Transgender and Gender-Nonconforming Persons: A Qualitative Study	Explore the perspectives of undergraduate nursing students nearing graduation on providing care to transgender and gender-nonconforming (TGNC) individuals	Qualitative descriptive exploratory design using semi-structured individual interviews	Undergraduate nursing students	Students reported limited TGNC knowledge, minimal curricular content, and uncertainty about providing affirming care.	Integrate TGNC-focused education and experiential learning opportunities into nursing curricula.
2	Bell et al.	Learning About Culturally Humble Care of Sexual and Gender Minority Patients	Describe teaching strategies to promote culturally humble care for sexual and gender minority patients.	Prelicensure nursing students engaged in a video and guided discussion; evaluated via online surveys.	Prelicensure nursing students	Students reported increased awareness and understanding of culturally humble care practices.	Incorporate culturally humble teaching strategies into nursing education to enhance care for sexual and gender minorities.
3	Burkey et al.	Infusing LGBTQ Cultural Competency Into Nursing Curriculum	Describe the integration of LGBTQ cultural competency into nursing education.	Development and implementation of online modules and avatar simulations; assessed through student feedback.	Undergraduate nursing students	Students found the modules cost-effective and beneficial for learning LGBTQ cultural competencies.	Utilize interactive simulations to enhance LGBTQ cultural competency in nursing curricula.
4	Burton et al.	Queering Nursing Curricula	Explore factors influencing LGBTQIA+ individuals' daily lives and their impact on healthcare encounters.	Literature review and analysis of existing nursing curricula.	Not specified	Identified gaps in nursing education regarding LGBTQIA+ health and the need for inclusive curricula.	Incorporate queer theory and LGBTQIA+ health topics into nursing education to address identified gaps.
5	Camp-Spivey	Simulation-Based Integration of Transgender Patient Care and CPR Competency for Prelicensure Nursing Students	Assess impact of transgender-focused simulation with CPR scenario on communication and clinical competence.	Simulation with prebriefing and debriefing; student evaluations of learning and confidence.	Prelicensure nursing students	Students reported increased confidence in communication, prioritization, and CPR skills; valued inclusive care content.	Integrate transgender-focused simulation to enhance inclusive communication and clinical competence.
6	Carmichael et al.	Effect of an Education Intervention on Nursing Students' Knowledge of and Attitudes Toward Caring for Transgender and Nonbinary People	Examine the effect of TGNB-focused health education on nursing students' knowledge and attitudes.	One-group pre/post design using the TKABS-HP survey.	Undergraduate nursing students.	Knowledge increased post-intervention; attitudes improved on the Sex and Gender Beliefs subscale.	Integrate TGNB health education into nursing curricula to strengthen gender-affirming care competencies.
7	Carney & Baser	Affirming Simulation in Nursing Education Across Different Institutional Contexts	Describe development and implementation of a TGNB-focused standardized-patient simulation across institutions.	Simulation with TGNB standardized patients, prebriefing, immersive scenario, and debriefing; reflective assessments.	Prelicensure nursing students.	Students demonstrated inclusive communication and affirming care skills; debriefing supported reflective learning.	Integrate TGNB-focused SP simulations into core curricula and use culturally humble, competency-based approaches.
8	Cassidy et al.	Stakeholders Inform an LGBTQIA+ Health Best Practices Learning Module for Nursing Students	Develop a learning module on LGBTQIA+ health best practices informed by stakeholder input.	Conducted focus groups with stakeholders; thematic analysis guided module development.	Nursing students and stakeholders from a large public university	Three themes emerged: appropriate terminology, health disparities, and respectful communication.	Integrate stakeholder-informed modules into nursing curricula to improve LGBTQIA+ health education.
9	Cole et al.	Empowering nursing students through inclusivity training for LGBTQIA+ patients: A quasi-experimental study	Assess effectiveness of an LGBTQIA+ inclusivity training module for baccalaureate nursing students.	Pretest and posttest surveys assessing GAP scores and LGBTQIA+ knowledge following a computer-based simulation module.	Baccalaureate nursing students.	Students showed significant improvements in affirmative practice attitudes and LGBTQIA+ knowledge after training.	Integrate structured LGBTQIA+ inclusivity training into undergraduate nursing curricula to improve preparedness and culturally responsive care.
10	Cox et al.	A guide to application of diversity, equity, and inclusion (DEI) principles for prelicensure nursing education	Describe integration of DEI principles in prelicensure nursing education.	Descriptive/Program development; No formal measures reported.	Prelicensure nursing students.	Students reported positive experiences with DEI-integrated coursework.	Integrate DEI content throughout nursing curricula using structured activities.
11	Day et al.	Hidden No More	Describe LGBTQIA+ content inclusion in nursing school lectures.	Lecture-based intervention with descriptive reporting.	Undergraduate and graduate nursing students	Inclusion of diverse identities broadened student knowledge of LGBTQIA+ health.	Expand lecture content to address comprehensive LGBTQIA+ health needs.
12	De Guzman et al.	LGBT inclusivity in health assessment textbooks	Assess LGBTQ inclusivity in health assessment nursing textbooks.	Content analysis; Reviewed textbook language and examples for inclusion.	Not applicable (textbook analysis).	Identified limited LGBTQ-inclusive content in health assessment textbooks.	Revise health assessment textbooks to reflect inclusive language and examples.
13	Elertson and McNiel	Answering the Call	Implement a multifaceted training on LGBTQ care using dialogue and reflection.	Surveys and journal reflections post-intervention.	Senior baccalaureate nursing students	Students showed increased empathy and engagement with LGBTQ care topics.	Use reflective methods and storytelling to deepen understanding of LGBTQ health.
14	Englund et al.	Using Simulation to Improve Students' Proficiency in Taking the Sexual History of Patients Identifying as LGBTQ: A Pilot Study	Evaluate impact of simulation on LGBTQ+ sexual history-taking skills.	Mixed methods; Standardized patient simulation with pre/post surveys and qualitative reflections.	Undergraduate nursing students.	Students reported increased confidence and communication skills in taking sexual histories from LGBTQ+ patients.	Use SP simulation to strengthen LGBTQ+ communication skills in sexual history-taking.
15	Gedzyk-Nieman et al.	Improving LGBTQ+ Health Equity via Nursing Education	Describe strategies to embed LGBTQ+ content into nursing curricula.	Review of educational tools including simulations and case studies.	Not specified	Various teaching tools can effectively build LGBTQ+ health awareness.	Use diverse teaching strategies to normalize LGBTQ+ health topics in education.
16	Hannans	Integrating LGBTQI+ Content in Nursing Education Using Immersive Virtual Reality	Evaluate VR simulation to improve empathy in LGBTQI+ care.	Facilitated discussions following 90-minute simulation experience.	Senior undergraduate nursing students	VR fostered empathy and recognition of LGBTQ health inequities.	Adopt VR simulations to support emotional learning around LGBTQ care.
17	Henriquez et al.	It's complicated: Improving undergraduate nursing students' understanding family and care of LGBTQ older adults.	Explore nursing students' knowledge and perceptions regarding inclusion of LGBT+ content in the nursing curriculum.	Descriptive cross-sectional survey	Undergraduate nursing students	Students reported limited knowledge of LGBT+ health content and perceived gaps in curricular coverage.	Integrate structured LGBT+ health content into undergraduate curricula to improve student preparedness and cultural competence.
18	Hickerson et al.	Sexual Orientation/Gender Identity Cultural Competence: A Simulation Pilot Study	Pilot a simulation to enhance LGBTQ+ cultural competence in nursing education.	Mixed methods; Cultural competence scale administered pre/post simulation, plus narrative responses.	Undergraduate nursing students.	Simulation led to measurable improvements in cultural competence scores and reflective insights.	Incorporate cultural competence simulations to support inclusive care training.
19	Jordan	Using a Flipped Classroom and Role-Play to Introduce Nursing Students to LGBTQIA+ Patient Care	Evaluate the effectiveness of a flipped classroom and role-play in teaching LGBTQIA+ patient care.	Implemented video-based self-instruction followed by in-class discussions and role-play; assessed via pre- and post-tests.	Prelicensure baccalaureate nursing students	Students reported increased knowledge and comfort in caring for LGBTQIA+ patients.	Adopt flipped classroom and role-play strategies to enhance LGBTQIA+ patient care education in nursing programs.
20	Karlin et al.	Development and Implementation of LGBT Simulations With Standardized Patients	Develop and test simulation scenarios for LGBTQ+ primary care.	Online modules and SP scenarios with pre/post testing.	Doctor of nursing practice students	Students reported improved clinical preparedness and comfort.	Use SP simulations to enhance LGBTQ+ communication and clinical skills.
21	Kerr et al.	Care of the Transgender Surgical Client: A Video Simulation for Baccalaureate Nursing Students	Describe development and use of a video simulation to support inclusive communication and care for transgender surgical clients.	Video simulation with facilitated classroom discussion.	Baccalaureate nursing students	Students were engaged and demonstrated improved understanding of inclusive communication with transgender clients	Incorporate video-based simulation to teach inclusive, gender-affirming care within undergraduate nursing curricula.
22	Klepper et al.	LGBTQI+ Representation in Pre-Licensure Nursing Textbooks	Evaluate LGBTQI+ representation in nursing textbooks.	Qualitative content analysis of pre-licensure nursing textbooks.	Not applicable	LGBTQI+ terms were inconsistently and minimally represented across textbooks.	Improve representation and accuracy of LGBTQI+ content in nursing textbooks.
23	Klotzbaugh et al.	Results and implications from a gender minority health education module for advance practice nursing students	Evaluate the effectiveness of a gender minority health module for advanced practice nursing students.	Mixed methods; Pre/post surveys with knowledge and attitude measures, plus open-ended responses.	Advanced practice nursing students.	Participants demonstrated increased knowledge and more affirming attitudes toward gender minority patients.	Implement targeted modules to enhance gender minority health education in advanced practice training.
24	Koch et al.	Role-Play Simulation to Teach Nursing Students How to Provide Culturally Sensitive Care to Transgender Patients	Evaluate role-play for teaching culturally sensitive transgender care.	Post-simulation survey following standardized patient scenario.	Accelerated second-degree BSN students	Students reported increased comfort using affirming language and navigating gender identity.	Incorporate role-play with SPs to prepare students for inclusive transgender care.
25	Kuzma et al.	Improving lesbian, gay, bisexual, transgender, and queer/questioning health: Using a standardized patient experience to educate advanced practice nursing students	Evaluate a standardized patient experience to teach inclusive LGBTQ+ care.	Standardized patient simulation; Evaluated through pre/post surveys and reflective journaling.	Advanced practice nursing students.	Students reported improved preparedness and communication skills with LGBTQ+ patients.	Use SP experiences to promote affirming care practices in advanced nursing education.
26	Maley et al.	A writing assignment to address gaps in the nursing curriculum regarding health issues of LGBT+ populations	Address gaps in nursing curriculum through a reflective writing assignment on LGBT+ health.	Descriptive; Student essays and instructor feedback, with qualitative review.	Prelicensure nursing students.	Students demonstrated increased awareness of LGBT+ health disparities and care challenges.	Use reflective writing to promote critical thinking about LGBTQ+ health equity.
27	Martin Walker et al.	The Use of Nursing Theory to Support Sexual and Reproductive Health Care Education	Describe use of nursing theory to support SGM health education.	Discussion and application of Caritas Processes to patient interaction.	Not specified	Theory-based approaches may reduce microaggressions in SGM care.	Apply Watson's Theory of Caring to improve affirming communication.
28	Maruca et al.	Using Simulation with Nursing Students to Promote Affirmative Practice Toward the Lesbian, Gay, Bisexual, and Transgender Population: A Multisite Study	Evaluate impact of simulation on affirmative practice toward LGBTQ+ patients across multiple sites.	Mixed methods; Standardized patient simulation, pre/post surveys, and qualitative feedback.	Prelicensure nursing students across three academic institutions.	Simulation improved students' confidence, skills, and empathy in LGBTQ+ care.	Use multisite SP simulations to build affirmative practice competencies in nursing education.
29	McElwain & Carr	Providing affirming care for LGBTQ patients	Promote affirming care practices for LGBTQ patients through education and reflection.	Descriptive; Integrated LGBTQ+ patient narratives and group discussions with reflective activities.	Undergraduate nursing students.	Students reported improved empathy and awareness of affirming care principles.	Incorporate storytelling and discussion to foster affirming attitudes toward LGBTQ patients.
30	McEwing	Delivering culturally competent care to the lesbian, gay, bisexual, and transgender (LGBT) population: Education for nursing students	Enhance cultural competence in caring for LGBT populations through targeted education.	Narrative review with case study examples and suggested curricular strategies.	Not applicable (curricular recommendations).	Identified need for structured LGBT content to build cultural competence in nursing.	Embed LGBT-focused scenarios and case studies in nursing education to improve competency.
31	McNeil et al.	Advocacy and Awareness: Integrating LGBTQ Health Education Into the Prelicensure Curriculum	Integrate LGBTQ health education into prelicensure nursing curriculum through advocacy and awareness.	Descriptive; Curriculum mapping and implementation strategies shared across academic settings.	Prelicensure nursing students.	Faculty and student engagement supported sustainable LGBTQ content integration.	Use curriculum mapping and stakeholder advocacy to embed LGBTQ content in nursing programs.
32	Mitchell et al.	Student Perceptions of a Novel Learning Method to Improve LGBTQ+ Knowledge	Assess student perceptions of a novel learning method for LGBTQ+ education.	Standardized patient simulation and pre/post surveys using SOCCS.	Graduate nursing students	Students reported improved understanding and intention to change practice.	Use standardized patient simulations to enhance LGBTQ+ cultural competence.
33	Muckler et al.	Transgender Simulation Scenario Pilot Project	Pilot a transgender patient simulation scenario to promote affirming care practices.	Standardized patient simulation; Pre/post surveys measuring comfort and communication.	Undergraduate nursing students.	Students reported improved comfort, preparedness, and language use in transgender care.	Include transgender-focused SP scenarios in clinical training to improve affirming care.
34	Mueller et al.	Measuring the Longitudinal Impact of a Transgender and Gender Diverse Curriculum	Assess impact of TGD curriculum on competence and attitudes.	Pre-post surveys using SOCCS v3 and Kirkpatrick-based post-tests.	Nurse practitioner students and faculty	Significant improvements in cultural competence from pre- to post-intervention.	Incorporate multimodal TGD curriculum to build competence in gender-diverse care.
35	Mueller and DeSimone	Bringing Gender-Affirming Care to Primary Care	Evaluate multimodal curriculum to educate NPs on gender-affirming care.	Pre/post survey using SOCCS v3 and qualitative reflections.	Nurse practitioner students	Students showed improved competence and confidence in transgender care.	Implement multimodal training on gender-affirming care in primary care settings.
36	Murphy	Implicit Bias Toward Lesbian and Gay Persons Among Nursing Students: A Correlation Study	Measure implicit bias toward lesbian and gay persons among baccalaureate nursing students.	Descriptive correlational design using the Sexuality Implicit Association Test and demographics.	Baccalaureate nursing students.	Students showed moderate implicit bias favoring straight over LG persons (*D* = 0.22). Male gender, straight identity, stronger religiosity, and RN-BSN program enrollment predicted higher bias.	Enhance curricula addressing sexual minority health, integrate implicit bias education, review programs for inclusiveness, and combine implicit and explicit measures in future research.
37	Ness et al.	Gender Affirming Postop Care Simulation for Prelicensure Nursing Students	Evaluate impact of SP simulation on gender-affirming postop care.	Pre/post survey using Park & Safer tool; narrative feedback.	Prelicensure nursing students	Students reported improved comfort and preparedness with affirming postop care.	Use SP simulation to build gender-affirming postop care skills.
38	Oosting et al.	Development and Implementation of an Asynchronous Online Interprofessional Course in LGBTQ+ Health	Develop and implement an asynchronous, interprofessional LGBTQ+ health course.	Descriptive; Asynchronous 15-week course with reflective assignments and student feedback.	Prelicensure nursing students.	Students reported satisfaction and perceived learning; formal data analysis pending.	Use asynchronous formats to improve access to LGBTQ+ health education and support interprofessional collaboration.
39	Ozkara et al.	The influence of the oncology-focused transgender-simulated patient simulation on nursing students' cultural competence development	Assess impact of oncology-focused transgender SP simulation on nursing students' cultural competence.	Mixed methods; Standardized patient simulation with pre/post surveys using validated cultural competence measures.	Undergraduate nursing students.	Students showed increased cultural competence and awareness of transgender oncology care needs.	Use SP simulation to enhance nursing competence in gender-diverse oncology care.
40	Ozkara et al.	Effect of the Diverse Standardized Patient Simulation (DSPS) Cultural Competence Education Strategy on Nursing Students' Transcultural Self-Efficacy Perceptions	Evaluate effect of Diverse Standardized Patient Simulation (DSPS) on transcultural self-efficacy.	Quantitative; Pre/post surveys using the Transcultural Self-Efficacy Tool.	Undergraduate nursing students.	Students demonstrated significant improvements in self-efficacy for cross-cultural care.	Incorporate DSPS strategies to strengthen transcultural nursing confidence.
41	Ozkara et al.	Transgender Standardized Patient Simulation: Management of an Oncological Emergency	Examine student response to transgender SP simulation for oncological emergency care.	Mixed methods; SP simulation with evaluation surveys and reflective feedback.	Prelicensure nursing students.	Students reported greater readiness and confidence in gender-affirming clinical scenarios.	Use transgender SP simulations to prepare students for complex, inclusive care delivery.
42	Paradiso et al.	Teaching and Learning About the Transgender Population	Assess whether a simulation experience improves students' knowledge and confidence in caring for LGBT older adults.	Simulation-based learning activity with pre/post evaluation of student knowledge, attitudes, and perceived preparedness.	Undergraduate nursing students.	Simulation increased students' understanding of LGBT older adults' health needs and improved comfort and preparedness in providing inclusive care.	Integrate LGBT-focused simulation experiences into nursing curricula to enhance culturally competent, affirming care for older adults.
43	Paradiso et al.	Integration of Transgender Health Using a Multimodal Approach	Integrate transgender health topics into nursing curriculum using multimodal strategies.	Pre/post tests, survey, and panel discussions; qualitative reflections collected.	Undergraduate nursing students	Students gained confidence and cultural awareness in transgender care.	Use layered teaching strategies to prepare students for affirming transgender care.
44	Pittiglio and Lidtke	The Use of Simulation to Enhance LGBTQ+ Care Competencies	Evaluate use of simulation to improve LGBTQ+ care competencies.	Pre/post simulation surveys adapted from GAP scale.	Undergraduate nursing students	Simulation increased comfort and skill with LGBTQ+ inclusive care.	Incorporate simulations with LGBTQ+ case scenarios in nursing education.
45	Rodhe and Goode	Using Simulation to Teach Gender-Affirming Care Concepts in Nursing Education	Examine whether simulation improves nursing students' knowledge and confidence in providing gender-affirming care to transgender and gender-nonconforming patients.	Low-fidelity simulation; prerecorded video with pre/post surveys assessing knowledge and confidence.	Prelicensure nursing students.	Increased understanding of transgender/GNC health needs and greater confidence in providing gender-affirming care.	Integrate low-fidelity simulation to introduce gender-affirming care concepts into nursing education.
46	Ruud et al.	Health History Skills for Interprofessional Learners in Transgender and Nonbinary Populations	Teach inclusive history-taking through standardized patient simulation.	Comfort, Skills and Attitudes in Transgender and Nonbinary Care Survey.	Midwifery, NP, and OB-GYN students and residents	Simulation improved learner comfort with history-taking for transgender patients.	Use interprofessional SP simulation to teach gender-affirming communication.
47	Safdar et al.	Integrating Sex and Gender into an Interprofessional Curriculum: Workshop Proceedings from the 2018 Sex and Gender Health Education Summit	Describe outcomes of an interprofessional workshop on integrating sex and gender in health education.	Descriptive; Post-workshop evaluations and expert consensus proceedings.	Interprofessional health educators and trainees.	Participants supported integrating sex and gender content across curricula.	Develop structured, interprofessional modules to address sex and gender in clinical training.
48	Schweiger-Whalen et al.	Converging Cultures: Partnering in Affirmative and Inclusive Health Care for Members of the Lesbian, Gay, Bisexual, and Transgender Community	Describe an academic-community partnership to support inclusive LGBTQ+ healthcare.	Descriptive; Partnership implementation with qualitative feedback from participants.	Nursing students and community-based healthcare providers.	Collaboration enhanced understanding of inclusive care and created shared learning opportunities.	Foster academic-community partnerships to advance inclusive LGBTQ+ education and practice.
49	Schwindt et al.	Just Another Patient? Student Reflections on Providing Mental Health Care to Transgender and Gender Expansive People During Simulated Encounters					
50	Sherman et al.	LGBTQ+ health education for nurses: An innovative approach to improving nursing curricula	Present an innovative educational model to improve LGBTQ+ nursing curricula.	Descriptive; Curriculum design and integration process with faculty review.	Undergraduate nursing students and faculty.	Faculty successfully embedded LGBTQ+ content with institutional support.	Combine faculty development with curriculum redesign to enhance LGBTQ+ nursing education.
51	Sherman et al. (1)	Development and Psychometric Properties of the Tool for Assessing LGBTQI+ Health Training	Develop and validate a tool to assess LGBTQI+ content in nursing curricula.	Expert panel review and face validity scoring of tool items.	Prelicensure nursing faculty and experts	Tool showed acceptable clarity, consistency, and content validity.	Use validated tools to evaluate LGBTQI+ content in nursing education.
52	Sherman et al. (2)	Transgender and Gender Diverse Health Education for Future Nurses	Evaluate a curriculum for improving TGD knowledge and attitudes.	Three-part online survey using SOCCS and attitude measures.	Accelerated BSN students	Students reported higher confidence and awareness in TGD care.	Integrate longitudinal TGD content across the nursing curriculum.
53	Smith et al.	An Interdisciplinary Approach to Enhancing Health Knowledge and Cultural Awareness With LGBT Older Adults	Evaluate an interdisciplinary simulation and panel to improve LGBT aging knowledge.	Pre/post surveys using validated perception and knowledge scales.	Nursing students and interprofessional faculty participants	Intervention improved comfort and knowledge working with LGBT older adults.	Include interprofessional simulation and panels to teach LGBT aging topics.
54	Steuben et al.	Implementing an LGBTQ+ interprofessional simulation with undergraduate nursing students	Pilot an interprofessional simulation to integrate LGBTQ+ care concepts into undergraduate curricula.	Interprofessional simulation; feedback from students, faculty, and standardized participants.	Undergraduate nursing students.	Participants perceived the scenario as valuable and effective for teaching LGBTQ+ care concepts.	Incorporate evidence-based, freely available simulation resources into nursing curricula to improve LGBTQ+ patient care competence.
55	Tartavoulle and Landry	Educating Nursing Students About Delivering Culturally Sensitive Care to Lesbian, Gay, Bisexual, Transgender, Questioning/Queer, Intersex, Plus Patients: The Impact of an Advocacy Program on Knowledge and Attitudes	Evaluate an advocacy-based educational program on LGBTQI+ culturally sensitive care.	Quantitative; Pre/post survey measuring knowledge and attitudes.	Undergraduate nursing students.	Significant improvements in knowledge and affirming attitudes were reported.	Use advocacy programs to build culturally sensitive care competencies in nursing students.
56	Teall et al.	Faculty Perceptions of Engaging Students in Active Learning to Address Implicit Bias Using Videos Exemplifying the Prenatal Visit of a Lesbian Couple	Explore faculty perceptions of using active learning to address bias in prenatal care.	Qualitative; Thematic analysis of faculty focus groups on video-based learning.	Nursing faculty and educators.	Faculty endorsed video exemplars as effective for bias awareness and student engagement.	Use active learning tools like clinical videos to support instruction on bias and LGBTQ+ care.
57	Tobbell	A Role for History in Inclusive Nursing Education	Explore the historical context of nursing's role in LGBTQ+ marginalization.	Narrative review and historical analysis.	Not applicable	Historical norms shaped systemic exclusion of LGBTQ+ people in care.	Include historical perspectives to contextualize equity in nursing education.
58	Vance et al.	A Pediatric Transgender Medicine Curriculum for Multidisciplinary Trainees	Evaluate pediatric transgender medicine curriculum for interprofessional trainees.	Mixed methods; Pre/post surveys, knowledge tests, and feedback from didactics and simulation.	Medical and nursing trainees in pediatrics.	Participants showed improved confidence and skills in pediatric transgender care.	Offer interprofessional training to strengthen pediatric transgender competencies.
59	Vance et al.	Using Standardized Patients to Augment Communication Skills and Self-Efficacy in Caring for Transgender Youth	Assess impact of SP encounters and e-learning on TGD youth care.	Pre/post e-learning modules and standardized patient evaluations.	Healthcare students caring for transgender youth	SP training improved communication skills and self-efficacy.	Incorporate SP encounters into TGD youth training for healthcare providers.
60	Velasco et al.	Nursing, Social Justice, and Health Inequities	Analyze Emancipatory Nursing Praxis as a framework for social justice education.	Theoretical analysis of nursing education literature.	Not applicable	ENP framework emphasizes critical reflection and structural change.	Adopt ENP to integrate social justice into nursing teaching, research, and policy.
61	Waxman et al.	Using Simulation to Provide Culturally Competent Care to Transgender and Gender Nonconforming Patients	Assess cultural competence gains from a transgender simulation scenario.	Mixed methods; SP simulation with surveys and qualitative reflection.	Undergraduate nursing students.	Students reported increased cultural sensitivity and inclusive communication.	Integrate simulation to develop affirming care skills for transgender patients.
62	White et al.	A sexual health course for advanced practice registered nurses: Effect on preparedness, comfort, and confidence in delivering comprehensive care	Evaluate impact of a sexual health course on APRNs' preparedness for LGBTQ+ care.	Quantitative; Pre/post surveys assessing comfort, confidence, and preparedness.	Advanced practice registered nurses (APRNs).	Course significantly improved participants' perceived preparedness and competence.	Include sexual health content to improve LGBTQ+ care delivery among APRNs.

### 2.2 Qualitative interviews

**Figure 1:**
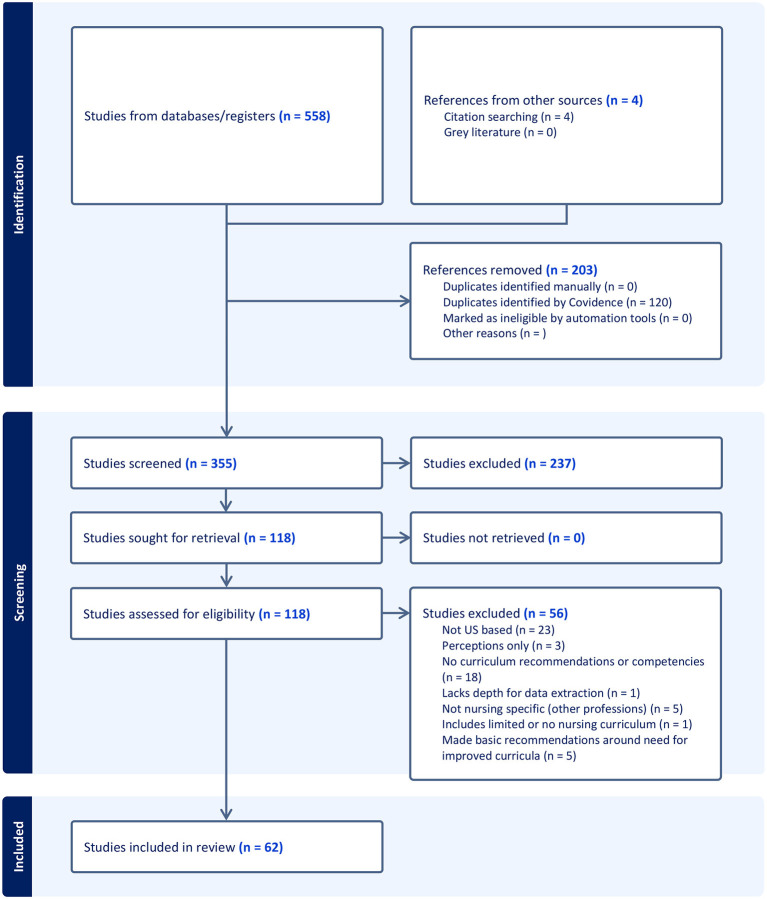
PRISMA flow diagram of study selection for the scoping review of U.S. nursing curricula.

To supplement findings from the systematic scoping review, we used a qualitative descriptive design, which is well suited for generating straightforward, practice-oriented insights from participant accounts. We conducted qualitative interviews and reported all Consolidated Criteria for Reporting Qualitative Research (COREQ) guidelines (11), summarized in Figure 1. The interviews were one-time, hour-long interviews (*n* = 21) with nursing students and faculty to garner additional perspectives on key themes. No individuals refused to participate, though one interviewee was unable to schedule a time and therefore was not included in the final sample. The interviewers included principal investigators (SM and KV) and students (NH) from our research team, either individually or as a team, representing a range of professional credentials (e.g., master's through doctoral education with training in qualitative methods) and personal credentials (e.g., lived experience as a sexual and/or gender minority, person of color). We had existing relationships with several participants through professional networks and associations, facilitating their willingness to be recruited into the study. Each interview was guided by an existing interview script and began with a brief description of the study goals as they related to our effort to contextualize scoping review findings on LGBTQ+-focused course content, delivery, and evaluation.

Participants were recruited using purposive sampling to capture perspectives from nursing faculty engaged in LGBTQ+ curriculum development and implementation. IRB-approved recruitment flyers were posted in a local primary care clinic specializing in LGBTQ+ care as well as in the campus Student Center. During an all-school meeting, School of Nursing faculty were informed about the project and invited to participate in scheduled focus groups. Recruitment materials were also emailed to leaders of local minority nursing associations (Black Nurses Association, Men in Nursing, National Association of Hispanic Nurses, and the Philippine Nurses Association of America) with a request to share the flier with their members. Additional participants were identified through snowball sampling, and eligible faculty with teaching experience were approached both in person and via email.

Participants selected a focus group time that best fit their schedules and received an information sheet in advance. On the day of the session, the information sheet was reviewed again, and participants were given the opportunity to ask questions before the 1-h discussion began. Each participant received a $20 appreciation incentive.

Interviews lasted 30–60 min and were conducted via HIPAA-compliant Zoom, recorded for research-team use, and supplemented with field notes. Interview guides were designed to contextualize findings from our scoping review, and thematic saturation was determined through ongoing discussion during monthly team meetings.

Our qualitative analysis used a multi-step rapid content analysis process (12). To enhance rigor, the team used consensus coding, maintained an audit trail, and engaged in iterative discussion to ensure analytic consistency. Two study team members coded the data, with consistency checks from the broader team. An excel matrix corresponding to the interview guide questions was created to summarize interview content (e.g., what are the most and least effective teaching approaches, what feedback do they have on the core findings from the scoping review like the importance of teaching theories and approaches, terms and concepts); basic demographics of participants were included so that we could explore if differences within themes (e.g., differences based on interviewee being in a protective vs. a punitive policy environment). The matrix content was discussed with the study team and with other key stakeholders (e.g., individuals involved in developing the subsequent course based on the scoping review and qualitative interview findings), but not with participants themselves to agree on key themes and considerations to be addressed in creating the course. Key quotations illustrating the consistency between the major themes, as well as some minor but still interesting themes, are included in Table 4.

**Table 4:** Quotations.

**Key theme**	**Quote**
**Part 1: course content**	
Theories and frameworks	“Once you fully understand the importance of giving this care... there's some people, colleagues that I know that are... against even providing this care from, like, a religious standpoint. So, I think if we put those frameworks in it, it will align them. We'll understand it's not about a certain group doing certain things, but the bigger picture, right? And why we need to really, really do this.”
Terms and concepts	“It's so important that we maintain a culture of when we know better, we do better. And that translates to all areas of improving patient care, including terminology. We may change again.”
Clinical considerations	“A needs assessment is good and then tailoring [the concepts] to [student] needs, and then being more supportive and realistic about it, because if we can engage them, in understanding it they can take better care of the patients and the population.”
**Part 2. Course delivery considerations**	
Modalities	“It's a very subtle integration around gender and sexuality and those assumptions and breaking down those assumptions, but nothing overt to not re-stigmatize the groups, so the person just happens to be gender queer in the simulation”
Course length	“I think it will depend on the modality that you decide to use. If you‘re going to use a video module, and it's any longer than an hour, you've lost me, right? So, I think chunking it will be really important.”
Instructors and partnerships	“In a perfect world, we would … have standardized patients or even faculty or guest speakers who this is part of their lived experience and can better speak to how they care for this population, or maybe some of the negative aspects they've experienced in the healthcare setting.”
Cost	“I think for university faculty, I don't think a cost should be attached to it. For anyone outside of that, I think it would be appropriate, because this is something that you're infusing in the curriculum that you want faculty to do.”
**Part 3. Experiential and simulation based pedagogies**	
Simulation-based learning approaches	“I particularly like the simulation I mentioned earlier, especially with the real participants from the community. They weren't given a script. They were prompted to tell their true stories. And I think that builds empathy and connection, and all that better than you could build in a script in a standardized patient, because they have such individualized experiences.”
Experiential learning activities	You can read all the Powerpoints, all the textbooks, all the papers in the world on trans people. But you're still not gonna have that full understanding until you actually talk with a trans person. Actually, you know, learn about us, learn about our lives. Learn that we're not the type of people that media makes us out to be. We're just everyday people looking to live life, and I think actually speaking to not only trans people, but queer people and having them talk about their experiences, talk about how they want to be treated would do a lot more to benefit us than any kind of PowerPoints, or textbook module.
Gender-affirming clinical skill development	“We asked real people who identify as LGBTQIA to come in and they were instructed very clearly to tell their own story. And so I feel like that, adds an extra layer of both empathy connection, to actually meet a trans person and to connect with them in that space, I thought was just really incredible for the student learners.”

## 3 Learning environment

The above methods were combined to develop a course designed for a university setting. The target audience involves teaching faculty at accredited schools of nursing. The faculty of the course, described in detail below, involves a hybrid of trained LGBTQ+ experts (both clinicians and research faculty) as well as people with lived experience. The learning objectives are to differentiate among terms that describe LGBTQ+ identities, describe the developmental concerns of LGBTQ+ community members across the lifespan, discuss the evolution of LGBTQ+ rights and their influence on health care policy in the United States, incorporate developmentally and culturally appropriate assessment strategies into the care of LGBTQ+ populations, diagnose and manage health concerns unique to transgender individuals and other members of the LGBTQ+ communities, effectively advocate for socially just health policy formation at the local, state, and national levels, use affirmative communication skills to build trusting relationships and promote health among members of LGBTQ+ communities, and describe evidence-based teaching strategies to build competence in caring for members of LGBTQ+ communities among student nurses and other health team members. The lessons learned will be informed by formal assessment of its feasibility and acceptability within 1 year of the course's implementation.

## 4 Results

Our multiple methods results fall into three broad domains: (1) Course Content (i.e., LGBTQ+-focused theories & framework, evolving terms and concepts, empirical evidence of health inequities, and clinical considerations); (2) course delivery considerations (i.e., balancing hybrid modalities, varying lengths, balancing personal and lived experience among instructors, minimal costs, and validated approaches to course evaluation); and (3) experiential and simulation-based pedagogies for affirming and inclusive clinical practice. Table 3 presents the studies informing the Course Content domain and provides a visual overview to support interpretation of the themes that follow. Figure 1 offers a synthesized visual summary of the studies contributing to the Course Delivery Considerations domain and complements the accompanying narrative analysis. Table 4 highlights the studies addressing Experiential and Simulation-Based Pedagogies and visually supports the thematic synthesis for that domain.

### 4.1 Domain 1. Course content

#### 4.1.1 Theories and frameworks

The literature review and qualitative interviews collectively underscored the importance of using diverse theories, including those addressing the unique challenges faced by LGBTQ+ individuals, as well as learning and teaching approaches (Table 1) (13–15). Some frameworks focused on power structures, racial and ethnic bias, social justice; others addressed social, behavioral and cultural factors (16, 17). The peer-reviewed literature highlighted learning and teaching approaches as well as nursing-specific approaches (18). Interview findings reinforced these themes by illustrating how faculty and students interpreted and applied these frameworks in practice, offering experiential perspectives that complemented the literature. Together, the literature identified foundational theoretical approaches, while the interviews illustrated how faculty and students translate these frameworks into practice, reinforcing their complementary roles in curriculum development.

#### 4.1.2 Terms and concepts

Consistent with the literature, interview participants emphasized that accurate terminology and respectful communication form the foundation of LGBTQ+-affirming practice, but their accounts further contextualized how these concepts play out during real clinical encounters (19, 20). A few articles and participants noted the importance of using terms and concepts inclusively, not in a way that is harmful or describes exclusive concepts (21). Participants and articles highlighted the importance of applying these topics correctly on intake forms when gathering a patient's history. The interviews underscored the importance of terms and concepts but expanded by acknowledging the dynamic nature of language within LGBTQ+ communities (and what to do when the wrong terminology is used). Further, participants emphasized the importance of creating an adaptable space for patients to describe what these terms mean to them.

#### 4.1.3 Empirical evidence of health inequities with attention to stigma and discrimination

The peer-reviewed literature and qualitative interviews consistently underscored the importance of teaching the role of history and how current systems and structures (re)produce health inequities at multiple levels (16, 17, 21–24). The importance of talking about how LGBTQ+ individuals demonstrate resilience and resistance was also emphasized. A strengths-based approach to healthcare was advocated, focusing on fostering the resilience of LGBTQ+ populations rather than concentrating solely on deficits and disparities (23, 25, 26). Interview narratives provided concrete examples that echoed the structural gaps identified in the literature.

Peer-reviewed literature as well as participants expanded significantly on the role of bias more generally at both the individual and structural level (27). Indeed, the discussion of bias (implicit, explicit, and bias without qualifiers) appeared 52 times across seven interviews, making it one of the most discussed topics. Participants repeatedly emphasized the need for self-reflection to identify our own internal biases and called for the meaningful integration of diverse LGBTQ+ experiences (discussed in greater detail below) into course content and delivery (28, 29). Participants also reflected on how structural factors such as racism, homophobia, and transphobia significantly influence LGBTQ+ health in general and their ability to teach LGBTQ+ health in their classrooms. Participants felt strongly that talking about the broader legal and policy environment should be part of the classroom experience, with concrete guidance on how best to advocate for their LGBTQ+ patients with their local and national lawmakers, and how to train future nurses to advocate for their LGBTQ+ patients.

#### 4.1.4 Clinical considerations

The articles reviewed suggest that opportunities for more inclusive clinical treatment arise at three separate occasions during an appointment. These occasions and examples of inclusive care options included physical assessments (e.g., organ inventory-based changes to a body to align with their gender); intakes (e.g., asking for pronouns); procedures and other clinical management opportunities (e.g., training on assisted reproductive technologies in gynecology) (30). The importance of avoiding unnecessary or invasive questions driven by curiosity rather than clinical necessity was emphasized. Interviews also noted how faculty and students must navigate sensitive topics like pronouns, organ inventories, and mismatched legal documentation with competence and empathy (14, 31). It was noted that it can be hard to navigate these sensitive topics in any context, but it is likely more complex in politically punitive settings, especially in the Deep South (25, 27, 28, 32–36).

### 4.2 Domain 2. Course delivery considerations

#### 4.2.1 Modalities

The peer-reviewed literature reported the use of both online and in-person webinars and workshop trainings (37–39) Articles consistently reported standardized patient simulation (SPS) with debriefing as the most effective approach (40–45); case-based learning and didactic materials were also frequently referenced (46–51). Only one article mentioned using an immersive virtual reality experience with a 360°, three-dimensional VR headset; this approach (though costly) could help create dynamic learning settings, especially for younger generations who are more technology-savvy (52). Most studies use a multimodal approach (53–56). Recommendations included recorded learning opportunities with built-in mechanisms to make them interactive (e.g., role play); reflection (e.g., assessing their own stereotypes and bias), and direct interaction with the community's LGBTQ+ population via panels and/or guest speakers or as SPS (23, 27, 57–62). The studies consistently underscored the need to collaborate with disciplines such as social work, medicine, and public health (39, 63). Survey participants confirmed the modalities noted in the peer-reviewed literature and expressed a preference for combining asynchronous and synchronous formats. Case studies, role-play activities, and patient simulations were most frequently mentioned. The importance of integrating LGBTQ+ people in SPS was emphasized, with attention to representing diverse core identities (e.g., age, race, and ethnicity). These findings aligned with interview reflections, which similarly highlighted the need to balance accessibility, technological capacity, and learner engagement when selecting delivery modalities.

#### 4.2.2 Course length

The peer-reviewed literature documented significant variability in the completion time for the LGBTQ+ curriculum, ranging from <1 h to over a year across the course of a nursing degree program. There is limited evidence to indicate what the right amount of time would be: One study found that a single 30-min in-service training was not enough to find statistically significant changes in attitudes; however, one 4-h training resulted in significantly more favorable views about LGBTQ+ patients (26, 64). Participants advocated for a thoughtful balance between time constraints, depth of content, and interactive opportunities, with modular approaches emerging as a favored structure for effective LGBTQ+-related education. Interview participants expanded on these patterns by describing how course timing influenced their ability to meaningfully absorb and apply content, reinforcing the literature's emphasis on thoughtful calibration of instructional time.

#### 4.2.3 Instructors and partnerships

Peer-reviewed studies emphasize the importance of collaborative teaching between LGBTQ+ community and faculty with training in LGBTQ+ health (33, 60). For people from the community, it meant recognizing that their experience was one of many experiences within the LGBTQ+ community; for individuals outside the community, it meant having significant training in LGBTQ+ health to ensure that they could appropriately speak to the evolving evidence base of best practices (64). The qualitative interviews collectively illustrate that while instructor type and lived experience play a critical role in delivery of LGBTQ+ curriculum, educators can enhance effectiveness through thoughtful preparation, collaboration with community members, and a commitment to ongoing learning.

Interview participants emphasized the role of partnerships—both with communities and across disciplines (58). Participants discussed including community partners in the curriculum while remaining cognizant of the demands we may be putting on communities (e.g., by paying them for their time, recognizing their experience as expertise). Collaboration with disciplines like social work and public health was urged to equip students to function effectively within diverse teams, promoting a holistic approach to care; thus, educating nurse educators to include other disciplines was beneficial for viewing nursing education from a holistic lens (39). These insights paralleled scoping review findings that emphasized the importance of involving community partners and multidisciplinary educators as a core component of effective LGBTQ+ training.

#### 4.2.4 Cost

The qualitative interviews highlighted a need to minimize costs when training faculty and staff. Many participants noted that they do not have the financial flexibility to pay for training themselves, relying instead on their institutions to cover the cost. Additionally, participants noted the need to meet Continuing Education Unit (CEU) requirements for NCLEX renewal and professional goals. They observed that if the training can be counted as a CEU, then their institution may cover the cost. Many institutions have a limit on spending for CEUs, so participants viewed anywhere from $0–$250 as a reasonable cost for the training.

#### 4.2.5 Evaluation

Participants suggested that evaluating a training course for faculty has been challenging in the past due to inconsistencies in participation. Participants suggested many evaluation criteria: the most popular were a self-paced course with quizzes, in-person discussion, and a training completion certificate (28, 29, 65–70).

### 4.3 Domain 3. experiential and simulation-based pedagogies

#### 4.3.1 Simulation-based learning approaches

The literature published, and especially over the last few years, highlights a marked expansion in the use of simulation to prepare nursing students for LGBTQ+-affirming practice, particularly when caring for transgender and gender-diverse patients (14, 71, 72). Articles described the use of prerecorded simulations, standardized patient encounters, interactive case scenarios, and structured debriefing to deepen students' understanding of inclusive communication and clinical sensitivity (14, 43–45, 71–73). These approaches were consistently associated with improved confidence, strengthened clinical judgment, and greater awareness of gender-affirming principles (44, 72). Studies emphasized that simulation provides a psychologically safe environment in which students can observe inappropriate interactions, reflect on implicit bias, and rehearse affirming behaviors without risk to patients (73, 74). Low-cost adaptations, such as video-based simulations and facilitated group discussion, were highlighted as accessible options for undergraduate programs seeking to expand LGBTQ+-focused training (62). Interview reflections supported these conclusions, underscoring that structured simulations helped learners operationalize gender-affirming principles beyond theoretical understanding.

#### 4.3.2 Experiential learning activities

Several studies underscored the value of experiential learning as a complement to traditional classroom instruction (23). These activities included reflective exercises, guided observation, service-learning collaborations, and structured opportunities for students to engage with LGBTQ+ communities in nonclinical settings (62). Authors noted that experiential approaches help students integrate course content with real-world perspectives, recognize the lived experiences of LGBTQ+ individuals, and build empathy through active engagement. Such learning experiences were often described as pivotal for helping students move beyond conceptual understanding toward a more holistic appreciation of the social, cultural, and structural forces shaping LGBTQ+ health. Reflection emerged as a central component, with multiple studies demonstrating that guided debriefing and self-examination deepened learning and supported the development of culturally responsive practice (29, 73). These experiential approaches complemented the structured simulations described earlier by promoting reflective skill-building in less formal learning environments.

#### 4.3.3 Gender-affirming clinical skill development

A growing subset of articles focused specifically on the development of gender-affirming clinical micro-skills. These studies highlighted targeted training in pronoun use, affirming communication techniques, intake interactions, gender-inclusive assessment practices, and strategies for navigating sensitive clinical conversations (73). Simulation-supported skills training was shown to enhance students' ability to apply gender-affirming principles in patient encounters and reduce discomfort associated with caring for transgender and gender-nonconforming individuals (44, 74). Findings emphasized that even brief, structured training sessions can meaningfully improve students' preparedness to provide respectful and competent care (26). Collectively, these studies illustrate a shift toward competency-based, practice-oriented approaches that aim to equip students with the clinical behaviors required for affirming, patient-centered nursing care. Together, these findings show how structured, experiential, and skills-focused approaches collectively build clinical competency while reducing learner discomfort, offering a coherent set of pedagogical strategies for LGBTQ+-affirming education.

## 5 Discussion

As federal and state landscapes shift toward “whole person health”, questions remain regarding how to address the needs of populations experiencing health inequities. Executive Orders released in 2025 suggest that certain subpopulations, including LGBTQ+ people, are no longer a priority population. Given the number of LGBTQ+ people across the United States, especially in the South, we are unlikely to “make America healthy again” without addressing the needs of LGBTQ+ communities. Structural changes are needed, engaging healthcare providers and specifically nurses who have the most touchpoints with patients. Our results offer a roadmap for educating nursing faculty to achieve the highest attainable standard of health for all.

Over the past several years, the nursing education literature has shown notable forward movement in preparing students to provide affirming care for LGBTQ+ populations. Recent studies demonstrate a clear expansion of transgender and gender-diverse curricular content in particular, with programs increasingly embedding gender-affirming care principles into foundational coursework rather than treating them as supplemental topics. Simulation-based pedagogy has also accelerated, particularly the use of transgender and gender-nonconforming standardized patients, prerecorded simulation scenarios, and structured debriefing models that invite self-reflection, cultural humility, and bias awareness (23, 43, 71, 72, 74). Many of these interventions emphasize inclusive communication, therapeutic engagement, and confidence-building, reflecting a shift from “competence” framed as knowledge acquisition toward competence as an integrated clinical, relational, and ethical practice (26, 44). Several newer studies additionally highlight the role of interprofessional simulation and team-based learning in strengthening gender-affirming care behaviors (45). Taken together, these developments suggest meaningful progress in both the breadth and sophistication of LGBTQ+-focused nursing education since earlier stages of the literature.

Despite these advances, important gaps remain. Few studies evaluate long-term retention of LGBTQ+-affirming skills or examine whether educational gains translate into sustained practice change in clinical settings. Most interventions emphasize transgender care specifically, leaving comparatively limited attention to sexual minority health needs, intersectional identities, and barriers faced by older adults, rural communities, and racially minoritized LGBTQ+ populations. Measurement approaches also vary widely, with few validated tools available to assess communication, attitudes, or behavioral outcomes, and even fewer studies incorporating patient-reported experiences. Future research would benefit from longitudinal designs, rigorous evaluation frameworks, and attention to how nursing programs can embed LGBTQ+ health content across curricula rather than relying on one-time exposures. Continued work is also needed to co-design educational interventions with LGBTQ+ communities themselves, ensuring that training efforts reflect lived experience and community priorities.

To advance the knowledge gained from our multiple methods research, we are currently developing a self-paced, asynchronous online course for nursing faculty who teach primarily pre-licensure students. The aim of the course is to increase faculty capacity to teach their students to provide LGBTQ+ affirming care. The focus is on pre-licensure students because registered nurses provide most of the bedside care and are more likely to be one of the first health care professionals whom a patient encounters. Initially, the course was planned with eight modules: historical and theoretical perspectives; basic terminology; physical health assessment; mental health concerns common to members of LGBTQ+ communities; common general health concerns; treatments to include pharmacology; subgroup differences and special considerations; and role of the health care provider. After developing the initial course outline, the team met to discuss potential challenges that could impact the success of the course given the current political climate, anti-DEI mandates, and open hostility toward LGBTQ+ communities. In January 2025 the American Nurses Association published a revision of the Code of Ethics for Nurses that underscored the need to develop an eighth module titled: Teaching Strategies for Student Nurses and Related Health Team Members. This module addresses how to communicate critical concepts to students whose values, beliefs, and behaviors are antithetical to the Code and are barriers to providing equitable care.

To bolster the course, we addressed competency-based education (CBE) via embedded quizzes to check knowledge and understanding of content. The course will conclude with an online simulation to test faculty knowledge and application of concepts. We will pilot the course with faculty who teach in the pre-licensure program. There will be two assessment points where data on the course's feasibility and acceptability as well as preliminary efficacy in advancing nursing faculty's knowledge and ability to teach LGBTQ+ focused content are assessed. Information from the sessions will be used for mid-course correction. End-of-course feedback will be used to refine the course for the second pilot cohort.

The complexity of teaching and researching LGBTQ+ health cannot be understated. In our home state of Alabama, an anti-diversity, equity, and inclusion legislation went into effect on October 1, 2024. This law made it illegal to select students based on core identities for training opportunities. However, it did not prohibit the teaching of concepts related to what have become known as “divisive concepts” (e.g., discussion of race and ethnicity, LGBTQ+ and other marginalized identities). Many core aspects of the legislation were consistent with existing tenets of higher education (e.g., that faculty would not force students to agree with them) (75). However, the dramatic shift in the state and federal political landscape has significantly undercut many efforts that remained in place even after our legislation went into effect. Although much remains unknown as legislative sessions are ongoing, the potential retribution for engaging with these concepts in both the teaching and research spheres is significantly shifting institutional support across the U.S. in higher education in general, and especially in deeply conservative states such as our own. During such times we must work to develop strategies that enable us to prioritize the health of our most vulnerable, such as our LGBTQ+ communities, and articulate (in our grants and publications such as this one) that we cannot achieve health equity for all without actively addressing and improving the health of those who we know have the poorest health. As we balance political and empirical priorities, we lean heavily on our commitment to science, and our collaboration with communities, to ensure that we persevere in advancing health equity for all.

Together, findings from the scoping review and interviews suggest several practical implications for nursing education. First, programs should incorporate LGBTQ+ focused theories, evolving terminology, empirical evidence of inequities, and clinical considerations into foundational coursework to ensure a consistent knowledge base across learners. Second, schools may strengthen implementation by balancing didactic content with experiential and simulation-based approaches that offer structured opportunities to practice gender affirming communication and clinical skills. Third, partnerships among faculty, community-based organizations, and LGBTQ+ community members may support sustainability and accountability as courses evolve.

Future research is needed to evaluate the effectiveness of these curricular strategies, identify which combinations of content and pedagogy produce the strongest learning outcomes, and explore how political and institutional contexts shape implementation. Additional work should also examine how these curricular components can be adapted for diverse educational settings, including community colleges, accelerated programs, and schools with limited resources. The three domains identified in this study offer a flexible structure for replication, allowing programs to tailor content while maintaining core commitments to inclusivity and evidence informed teaching.

## 6 Limitations

This study has several methodological limitations. First, the scoping review relied solely on PubMed for the literature search, which may have excluded relevant articles indexed in other databases. Second, the qualitative interviews involved a relatively small, purposive and snowball sample of nursing faculty and students who were engaged in or connected to LGBTQ+ curriculum development and implementation. This approach may have overrepresented individuals who already have interest, expertise, or institutional support in this area, and the findings may not fully capture the perspectives of educators or learners who are less engaged or who work in more constrained environments. Third, recruitment procedures may have introduced selection bias by attracting individuals with preexisting interest in LGBTQ+ health or curriculum development. Fourth, some interviewers and participants were members of the same academic community, and existing professional relationships may have influenced how openly participants discussed concerns or critiques, even though reflexivity practices were used during data collection. Finally, the broader socio-political environment surrounding LGBTQ+ health and education, including increasing legislative restrictions in some states, may have shaped participants' perceptions of feasibility, safety, and institutional support.

## 7 Acknowledgment of constraints

As the federal and state landscape shifts toward “whole person health,” significant questions remain regarding how best to address the unique needs of populations like LGBTQ+ people, who experience pervasive health inequities. In particular, the Executive Orders released in January 2025 (76) suggest that certain subpopulations, such as LGBTQ+ people, are no longer a priority population. Given the substantial number of LGBTQ+ people across the United States (2024 Gallup Poll shows 9.3% of the country's population identifies as LGBTQ+) (77), and especially in the South (home to the largest LGBTQ+ population across Census Bureau regions), we are unlikely to “make America healthy again” without addressing the needs of LGBTQ+ communities. To do so, structural level change is needed to make the promise of good health for all Americans a reality, engaging nurses, who have the most touchpoints with our patients.

## 8 Conclusion

Our results highlight a gap in the ability of the nursing profession to adequately teach care for all populations. However, we have drawn a clear roadmap for educating our nursing faculty, empowering them to deliver on our collective commitment to prepare the next generation of nurses. We must equip our students with the skills to provide evidence-based, high-quality care that allows all individuals to attain their optimal health.

## Data Availability

The raw data supporting the conclusions of this article will be made available by the authors, without undue reservation.
